# Use of a robotic station for full automation of the determination of organic matter in soil and fertilizers

**DOI:** 10.1155/S1463924694000222

**Published:** 1994

**Authors:** P. Torres, J. A. García-Mesa, M. D. Luque de Castro

**Affiliations:** Department of Analytical Chemistry Faculty of Sciences Univerity of Córdoba Córdoba E 14004 Spain

## Abstract

A fully automatic method for the determination of organic matter has been implemented using a robotic station. The overall process involves weighing, dilution, oxidation in very harsh working conditions (K_2_Cr_2_O_7_ + concentrated H_2_SO_4_) dilution, centrifugation and photometric monitoring of the Cr(III) formed. Batches of six samples are manipulated by the robot, which also calibrates and delivers the results of the sample determination, both in soil and fertilizers, as percentages of organic matter which require slightly different sample treatment. The precision and validation of the method have been established using both types of samples and the results obtained compare well with those of the method used for routine analysis in official agricultural laboratories.


Journal of Automatic Chemistry, Vol. 16, No. 5 (September–October 1994), pp. 183–186

### Use of a robotic station for full automation of the determination of organic matter in soil and fertilizers

**P. Torres, J. A. García-Mesa and M. D. Luque de Castro**

*Department of Analytical Chemistry, Faculty of Sciences, Univerity of Córdoba, E 14004 Córdoba, Spain*

*A fully automatic method for the determination of organic matter has been implemented using a robotic station. The overall process involves weighing, dilution, oxidation in very harsh working conditions ($K_2Cr_2O_7$ + concentrated $H_2SO_4$), dilution, centrifugation and photometric monitoring of the Cr(III) formed. Batches of six samples are manipulated by the robot, which also calibrates and delivers the results of the sample determination, both in soil and fertilizers, as percentages of organic matter which require slightly different sample treatment. The precision and validation of the method have been established using both types of samples and the results obtained compare well with those of the method used for routine analysis in official agricultural laboratories.*

### Introduction

The decomposition of plant and animal remains in soil is a basic biological process during which carbon is recirculated to the atmosphere as carbon dioxide, nitrogen is made available mainly as ammonium ion and also as nitrate, and other associated elements such as phosphorus, sulphur and various micronutrients, appear in different forms required by plants. Some of the carbon is assimilated into microbial tissue and part is converted into stable humus and some native humus is mineralized. The total organic matter content is maintained at a steady-state level dependent on the soil and management system.

Soils vary greatly in organic matter content: from less than 1% in sandy soil to more than 60% in peat moss. A series of soil-forming factors determines the organic matter content, namely: climate, vegetation, topography, parent material age, and also the type of crop if the soil has been under cultivation.

Humus plays an important role in the physical and biological characteristics of a soil. Thus, such physical properties as texture, colour, and the capacity to retain water, are modified by the humus content. The amount of organic matter influences the formation of chelates with metal ions such as Cu(II), Mn(II) and Zn(II) (micronutrients), the pH, exchange capacity and the mineralization rate (for example, the availability of nitrogen, phosphorus and sulphur). It is also a factor in bioactivity and possibility of degradation of organic molecules, as pesticides [1].

Determining the percentage of organic matter in soil is therefore routinely analysed in agricultural laboratories. Two general methods are used, both of which are based on the oxidation of the organic matter: (*a*) dried methods, based on the measurement of the $CO_2$ released in the total combustion; and (*b*) wet methods, which use a dissolved oxidant. The degree of oxidation in the latter depends on the working conditions; so a statistical factor is often used—the value of this is calculated from the dried method. The oxidizing agent is usually potassium dichromate and the method is based on the reduction of Cr(VI) in an acid medium:

$$Cr_2O_7^{2-} + 14H^+ + 12e \quad 2Cr^{3+} + 7H_2O;$$

and on the oxidation of the organic matter, which acts as a carbohydrate:

$$C_2H_{12}O_6 - 24e \quad 6CO_2 + 6H_2O.$$

The amount of $Cr^{3+}$ formed, proportional to the humus present, can be estimated by a back titration with a ferrous salt [2], or directly by monitoring the absorbance at 590 nm.

These methods have a series of shortcomings, for example they are slow, they require a high degree of human participation, and the reagents are corrosive. These methods are frequently used in agricultural laboratories and so automation is highly desirable. This paper describes a fully automated method for the determination of organic matter, both in soil and fertilizer, using a robot for a number of processes—weighing and centrifugation, for example.

Methods for determining other parameters in soil have been previously reported, namely: for volatile compounds [3], pesticides [4], pH [5], conductivity [6] and phosphorus [7].

### Experimental

### *Reagents*

An aqueous 10% solution (w/w) $K_2Cr_2O_7$ was used as oxidant, together with concentrated $H_2SO_4$ containing 25 g/l $Ag_2SO_4$. The stock solution for standardization was 0·235 M $Na_2C_2O_4$, from which diluted solutions were prepared as required by addition of distilled water.

The reagents used for the development of the official method were 1 N $K_2Cr_2O_7$ + concentrated $H_2SO_4$ containing 25 g/l $Ag_2SO_4$, or chromic mixture, for soils and fertilizers, respectively. Concentrated $H_3PO_4$ or NaF were also used. The titration step was performed using aqueous 0·5 M ammonium ferrous sulfate (Mohr salt), containing 20 ml of concentrated $H_2SO_4$ per litre. Diphenylamine in sulphuric medium was used as indicator. All chemicals used were analytical reagent grade and were supplied by Merck.

0142-0453/94 $10 00 © 1994 Taylor & Francis Ltd

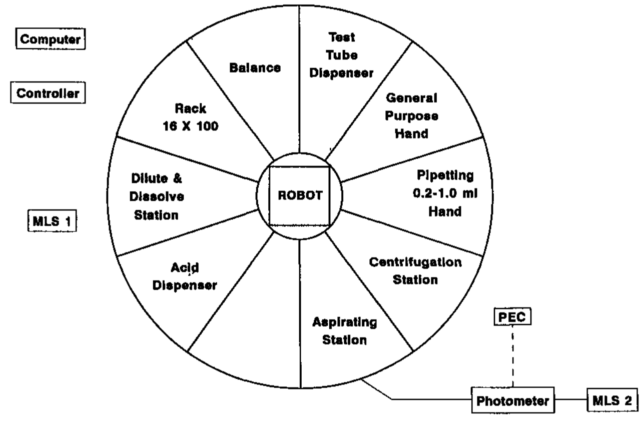

*Figure 1. Robotic station for implementation of a fully automatic method for determination of organic matter in soil and fertilizer.*

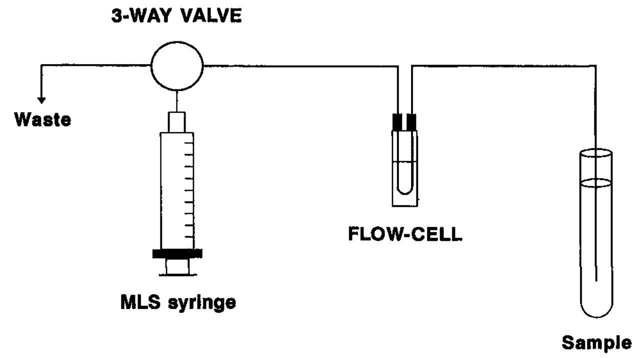

*Figure 2. Scheme of the use of the master laboratory syringe to pass the sample through the flow cell for monitoring.*

*Instruments and apparatus*

Instrumentation included a robotic station consisting of a Zymate II plus robot, a System V controller, a printer, an all-purpose hand, a syringe hand with a disposable pipette tip rack, a test tube dispenser, a 16 × 100 mm tube rack, a Mettler AE 200 balance, two Master Laboratory Stations (MLS), a Power & Event Controller (PEC), a dilute & dissolve station, a Z710 centrifuge, a Brand Dispensette liquid dispenser adaptable to a bottle, and a Unicam 8625 spectrophotometer connected to the AD converter of the PEC through its analogue output. The system V controller was interfaced to a Netset 286/400 personal computer (see figure 1).

*Manual procedure*

*Determination in soil:* 10 ml of 1 N $K_2Cr_2O_7$ are mixed with 0·5–1·0 g of sample, and 20 ml of $H_2SO_4$ containing 25 g/l $Ag_2SO_4$ are added slowly while the solution is stirred. The mixture is allowed to stand for 30 min on an asbestos plate. Then, the mixture is diluted with *c.* 200 ml distilled water, allowed to cool and 10 ml of concentrated $H_3PO_4$ are added. Afterwards the solution is titrated with Mohr salt solution (diphenylamine as indicator).

The addition of $Ag_2SO_4$ deals with avoiding the interference from chloride ions; and that of phosphoric acid (or sodium fluoride) with favouring the titration step by complexing the Fe(III) formed.

*Determination in fertilizer:* 10 ml of chromic mixture are mixed with 0·1–0·5 g of sample (the amount of sample depends on the foreseeable humus content). After smooth stirring the slurry is plunged into a boiling water bath for 90 s. A new stirring step is followed by a 5 min stand, and the heating/stirring/stand sequence is repeated three times before standing to allow for cooling for 30 min at room temperature.

The slurry is transferred to a 250 ml volumetric flask and made up to volume with distilled water. A 30 ml aliquot is diluted to *c.* 200 ml, 2 g of NaF, the indicator is added and the mixture is titrated with the Mohr salt solution.

Both manual procedures require a blank assay.

*Robotic procedure*

The robot performs the following operations: weighs a test tube, and *c.* 0·3 g of soil or 0·1 g of fertilizer. Then it adds 5·5 ml of 10% $K_2Cr_2O_7$ and 8 ml of $H_2SO_4$. After allowing the sample to stand for 30 min (during this interval the robot prepares five more samples), the robot adds distilled water to the test tube (from 2 to 9·5 ml for soil samples and from 0·5 to 9·5 ml for fertilizers), centrifugates and puts it in the aspiration tube of the flow-cell (see figure 2). The syringe aspirates the sample through the flow-cell where the absorbance is monitored at 590 nm in a dynamic regime. The absorbance data are acquired and treated by the computer. The robot also prepares and transports the solutions to run the calibration graph, which is stored by the computer.

### Results and discussion

The robot performs the first part of the method with dried and sieved samples: weighing the sample, and placing it in a test tube. As the all-purpose hand does not allow powder samples to be weighed, the test tube holder of the balance was altered to permit the top of the test tube where the sample was to be dispensed stand out of the balance [5]. Thus, the robot seized the tube containing the sample (sample container) and tipped it over the test tube in the balance. In this position, the bottom of the sample container came into contact with a small vibrating motor (whose go/stop sequence was controlled by the PEC through a switch closure) which worked over a preset interval, so an amount of sample, close to but smaller than that required for the determination, was dropped into the reception tube. Then, smaller additions of the sample were made with short working intervals of the vibrating motor until the sample amount was in the range selected depending on whether it was a soil or fertilizer.

Once the sample has been weighed, taking into account the capacity of the conventional test tubes, 5·5 ml of oxidant and 8 ml of $H_2SO_4$ were added to it. As the acid used was concentrated, it was not possible to use a syringe from the MLS for this, because of the acid attack. The use of a displacement bottle was assayed by helping the displacement with pressurized air to avoid the use of a selecting valve. Corrosion of the outlet tube and irreproducibility of the delivered volume meant that this approach was rejected. The corrosion was not the main shortcoming, but, rather, the irreproducibility of the volume of acid added which was a key to achieve precision of the monitored final solution, as the absorbance measured depended on the aqueous solution/sulphuric acid ratio (losses due to heating in the water/sulphuric acid mixture). Both problems were solved by using a commercial dispenser, which was easy for the robot to use, and could be adapted to the bottle of acid. A support for the reception test tube was also built, as well as two programs to command the robot and the peripherals.

After the addition of the oxidant and the acid, the test tube was allowed to stand in the rack for at least 30 min for total development of the oxidation reaction. During that interval the robot prepared five more samples; so the robot worked with batches of six samples—the maximum capacity of the centrifuge.

After the standing interval a 2 to 9·5 (for soil samples) or a 0·5 to 9·5 (for fertilizer samples) water dilution was performed. The samples were then centrifugated to deposit some suspended particles which could disturb the absorbance measurement.

The last step was the measurement of the absorbance at 590 nm to quantify the Cr(III) formed. There was no interference from the $Cr_2O_7^{2-}$ at this wavelength. The supernatant of the centrifuge tube was aspirated by an MLS syringe through a Teflon tube which kept vertical with the aid of a rigid support (figure 2). The measurement was performed in a dynamic regime and a small volume of air was aspirated between consecutive samples. There was no cross-contamination between samples; so a washing or rinsing step was unnecessary. The absorbance value of each sample was acquired by the computer and, after interpolation in the calibration graph, the result was expressed as the percentage of organic matter in the sample.

### *Calibration*

Before starting the sample manipulation, the robot prepared the standards and obtained the calibration graph. The Cr(III) standards could not be obtained by dissolution of a Cr(III) salt, but from $Cr_2O_7^{2-}$ and a reducing agent in the same working conditions as in the soil and fertilizer samples. Sodium oxalate was selected as the reductant and its oxidation product ($CO_2$) did not interfere with the Cr(III) monitoring.

The robot, through the MLS, poured five different aliquots of a 0·235 M sodium oxalate standard solution into test tubes, made them up to 5·5 ml with 10% $K_2Cr_2O_7$ and added 8 ml of $H_2SO_4$ solution. The tubes were allowed to stand for 30 min. Then, the same dilution as in the samples was performed, as well as the absorbance measurement. From these five standards the following equation was obtained:

$$A = 5{\cdot}3 \times 10^{-3} + 3{\cdot}24 \times 10^{-2}X \quad (r = 0{\cdot}9986)$$

where $A$ (in absorbance milliunit) is expressed as a function of the initial oxalate milliequivalent ($X$) and $r$ is the correlation coefficient.

*Table 1. Repeatability of the proposed method.*

| Soil | | Fertilizer | |
|---|---|---|---|
| Sample | % Organic matter | Sample | % Organic matter |
| 1 | 1·9 | 1 | 20·5 |
| 2 | 1·8 | 2 | 24·3 |
| 3 | 1·8 | 3 | 26·5 |
| 4 | 1·8 | 4 | 24·0 |
| 5 | 1·7 | 5 | 26·5 |
| 6 | 1·8 | 6 | 26·1 |
| 7 | 1·8 | 7 | 21·6 |
| 8 | 1·8 | 8 | 22·0 |
| 9 | 1·8 | 9 | 28·6 |
| 10 | 1·9 | 10 | 23·9 |
| 11 | 1·9 | 11 | 25·2 |

Mean: 1·8; R.S.D.: 3·3%. Mean: 24·5; R.S.D.: 9·9%.

*Table 2. Percentage of organic matter by the robotic and manual methods.*

| Sample | Robotic method | Manual method |
|---|---|---|
| Soil 1 | 3·6 ± 0·1 | 3·7 ± 0·1 |
| Soil 2 | 1·1 ± 0·0(3) | 1·1 ± 0·0(4) |
| Soil 3 | 0·8 ± 0·0(2) | 0·7 ± 0·0(2) |
| Soil 4 | 5·7 ± 0·1 | 6·2 ± 0·2 |
| Soil 5 | 1·5 ± 0·0(2) | 1·3 ± 0·1 |
| Soil 6 | 1·8 ± 0·0(6) | 1·4 ± 0·1 |
| Compost 1 | 24·5 ± 2·4 | 20·5 ± 0·7 |
| Compost 2 | 21·6 ± 0·4 | 29·9 ± 5·5 |

The numbers in brackets are not representative, but orientative.

### *Features and validation of the method*

The repeatability of the method was evaluated using 11 samples of the same soil and 11 of fertilizer. The results obtained are summarized in table 1.

The validation of the method was performed by comparing the results provided by the reported method and those from the official method [2] on 11 soils of different features and two commercial fertilizers (Compost). Table 2 shows the results of this study. A good agreement between the results and similar repeatability (expressed as r.s.d.) can be observed. The determinations were performed in quadruplicate in all instances.

The sampling frequency achieved was 4·5/h.

### Conclusions

A fully automated method for the determination of organic matter in soils and fertilizers has been reported. As the routine determination of organic matter is time-consuming, requires a high degree of human participation and makes use of corrosive substances, automation was considered an important goal. Since operations such as weighing, addition of corrosive reagents and centrifugation are involved in the overall process, full automation could only be achieved with a robotic station.

The problems involved in the use of corrosive substances

(i.e. concentrated sulphuric acid) have been overcome with a simple device very common in analytical laboratories.

### Acknowledgement

Dirección General de Investigación Cientifica y Técnica (DGICyT) is thanked for financial support.

### References

1. Stevenson, F. J., *Humus Chemistry* (Wiley Interscience, New York, 1982).
2. Ministerio de Agricultura, *Pesca y Alimentación, Métodos Oficiales de Análisis*, Vol. III (1986).
3. Makelar, M., Antloga, M. and Schmidt, S. A., In *Advances in Laboratory Automation Robotics*, Eds Strimaitis, J. R. and Hawk, G. L. (Zymark, Hopkinton, 1985).
4. Goodwing, P. A., In *Advances in Laboratory Automation Robotics*, Eds Strimaitis, J. R. and Helfrich, J. P. (Zymark, Hopkinton, 1990).
5. Torres, P., García-Mesa, J. A., Luque de Castro, M. D. and Valcárcel, M., *Fresenius' Zeitschrift für analytische Chemie*, **346** (1993), 704.
6. Torres, P., García-Mesa, J. A., Luque de Castro, M. D. and Valcárcel, M., *Laboratory Robotics Automation* (in press).
7. Torres, P., García-Mesa, J. A., Luque de Castro, M. D. and Valcárcel, M., *Laboratory Robotics Automation* (in press).

